# A novel injury mechanism of trans-styloid palmar-divergent dislocation of the lunate and scaphoid: a case report

**DOI:** 10.3389/fsurg.2025.1526008

**Published:** 2025-04-04

**Authors:** Zhiling Wang, Junjie Qu, Le Kang, Jianqiang Cui, Yanchao Wang, Chengli Li, Lin Xu

**Affiliations:** Department of Orthopaedics, Yantai Affiliated Hospital of Binzhou Medical University, Yantai, Shandong, China

**Keywords:** scapholunate dissociation, palmar-divergent injury, scaphoid dislocation, lunate dislocation, ORIF

## Abstract

**Background:**

Palmar-divergent dislocation of the lunate and scaphoid is a rare high-energy trauma with complex injured mechanism. Early diagnosis and surgery are necessary, however, there is no consensus on surgical treatment, as only a few literatures have been reported.

**Case summary:**

A 56-year-old man, following a powerful strike to the dorsal side of left wrist and forearm, complained about swelling, tenderness, and limited motion of the left wrist during the physical examination. According to physical and imaging results, this patient was diagnosed with trans-styloid palmar-divergent dislocation of the lunate and scaphoid, as well as radial diaphysis fracture. We performed open reduction, internal fixation with K-wires, ligament reconstruction, and cast immobilization for 6 weeks, followed by early rehabilitation.

**Conclusion:**

We firstly described a novel injury mechanism of trans-styloid palmar-divergent dislocation of the lunate and scaphoid based on the classical Mayfield's mechanism, and reviewed suggestions regarding the optimal treatment of palmar-divergent dislocation of the lunate and scaphoid.

## 1 Introduction

Lunate and scaphoid dislocation is a rare high-energy traumatic injury involving pronation and hyperextension of the wrist. The association of dislocation of lunate and scaphoid is few, and it is classified into two types: dislocation as a unit and divergent dislocation (1). Because of the potential short- and long-term complications including median nerve injury, carpal instability, traumatic arthritis or avascular necrosis (2), early diagnosis and treatment are essential to restore the anatomical structure of the wrist and prevent carpal instability (3). Currently, most surgeons recommend emergent closed/open reduction and internal fixation, and ligament reconstruction (4, 5). Herein, we firstly report a new case of trans-styloid palmar-divergent dislocation of the lunate and scaphoid complicated by radial fracture caused by a special injury mechanism, which was treated with open reduction and internal fixation (ORIF), ligament repair, and early functional rehabilitation. The objectives of this article are to discuss the injury mechanism and operative treatment of trans-styloid palmar-divergent dislocation of the lunate and scaphoid to better understand this injury. We hope this case contribute to a systematic review to establish a consensus on treatment, particularly regarding the restoration of wrist function.

This manuscript follows CARE guidelines. Written informed consent was obtained from the patient in this study for the publication of corresponding case information and images.

## 2 Case report

**Figure 1:**
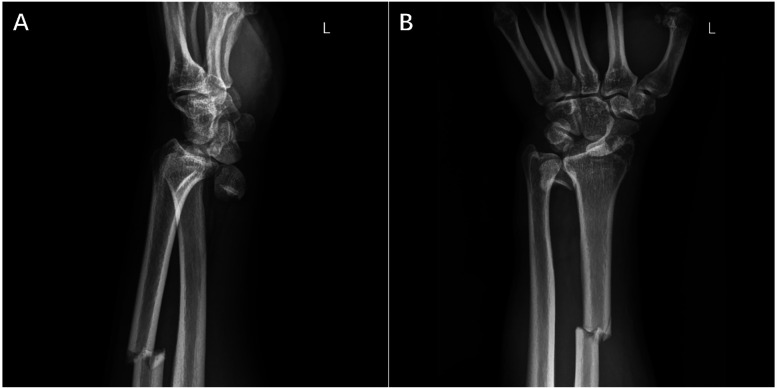
Pre-operative radiography nnshowed the injured left forearm. Lateral **(A)** and anteroposterior **(B)** radiographs of the left forearm showed dislocation of lunate and scaphoid as well as multiple radial fractures.

**Figure 2:**
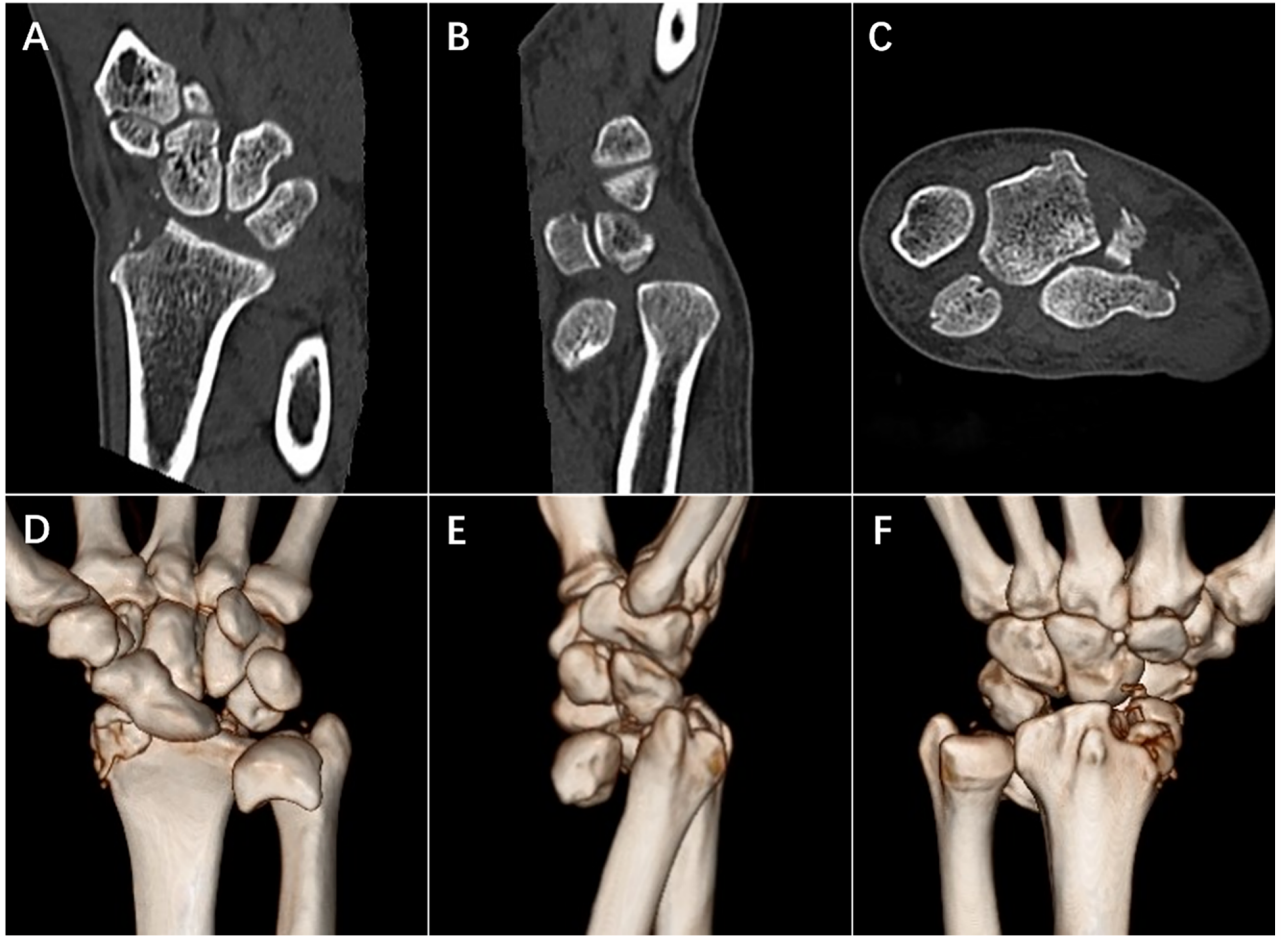
Pre-operative CT of injured left forearm. Coronal plane **(A)**, sagittal plane **(B)** and transverse plane **(C)** of CT imaging showed dislocation and separation of lunate and scaphoid as well as multiple fracture. 3D reconstruction results **(D**–**F)** showed injury in details.

A 56-year-old male worker presented to the Emergency Department of Yantai Affiliated Hospital of Binzhou Medical University after a strike to the dorsal side of his left wrist and forearm. The patient complained of severe pain and limited movement of the left wrist. The left wrist and forearm were swollen, with an obvious bulge on the volar side of the distal forearm, and left hand was hypoesthesia with limited motion, while the vascular status of the hand and forearm remained relatively normal. Radiographs and computed tomography (CT) showed lunate and scaphoid dislocation, scapholunate dissociation, radial styloid fracture as well as radial diaphysis fracture, and especially the lunate extruded into the forearm (Figures 1, 2). Some avulsed fragments were seen at radio-dorsal side of the scaphoid in CT image. The manual reduction of the lunate and scaphoid failed, and the patient was immediately scheduled for open reduction under brachial plexus nerve block.

**Figure 3:**
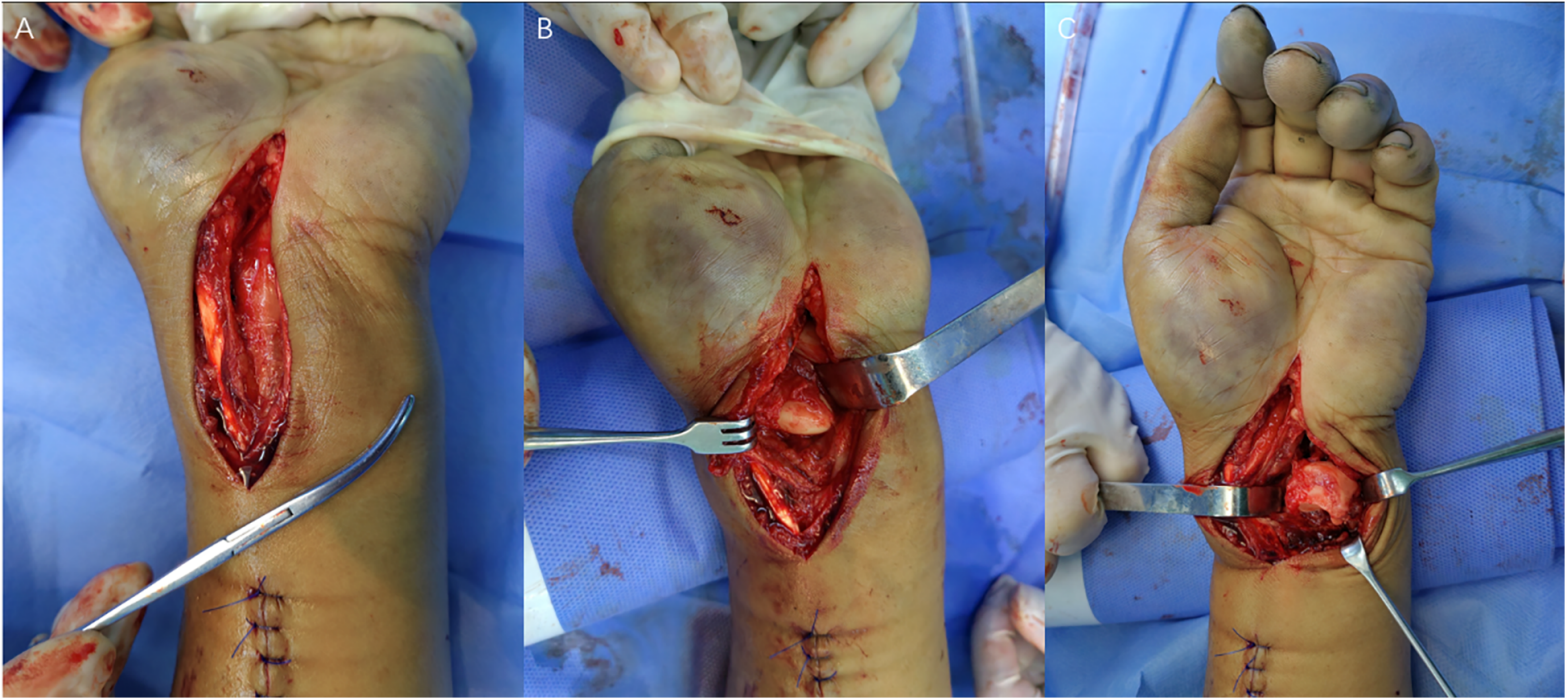
Intraoperative photos. **(A)** Median nerve exposure and decompression. **(B)** Scaphoid dislocation and disappeared lunate. **(C)** Isolated lunate in intermuscular septum.

The arm was kept in 90° abduction, and the radial diaphysis fracture was treated first with conventional open reduction plate internal fixation. Then, a 7 cm Z-shaped incision was made from the median of the palm to the proximal end, and the subcutaneous tissue and transverse carpal ligament were incised. The median nerve protruded forward, and the patient underwent median nerve decompression. A palmar dislocation of the scaphoid was detected, and the lunate fossa was empty, with the lunate missing. According to fluoroscopy, after separating the soft tissue toward the proximal end, the isolated lunate bone was found in the intermuscular septum between the flexor carpi ulnaris and the superficial flexor tendon of the fifth finger. The lunate and scaphoid were first reduced into their fossae. (Figure 3) The scapholunate (SL) joint was fixed using two 1.5 mm K-wires to maintain reduction (from the scaphoid to lunate crosswise). The scaphoid and lunate were fixed with the distal row of carpal bones using two K-wires (from radius to capitate and from radius to triquetrum bone). Two K-wires were inserted to fix the radial styloid fracture. The SL and lunotriquetral (LT) ligaments were sutured. Following these procedures, the entire forearm was fixed in plaster.

**Figure 4:**
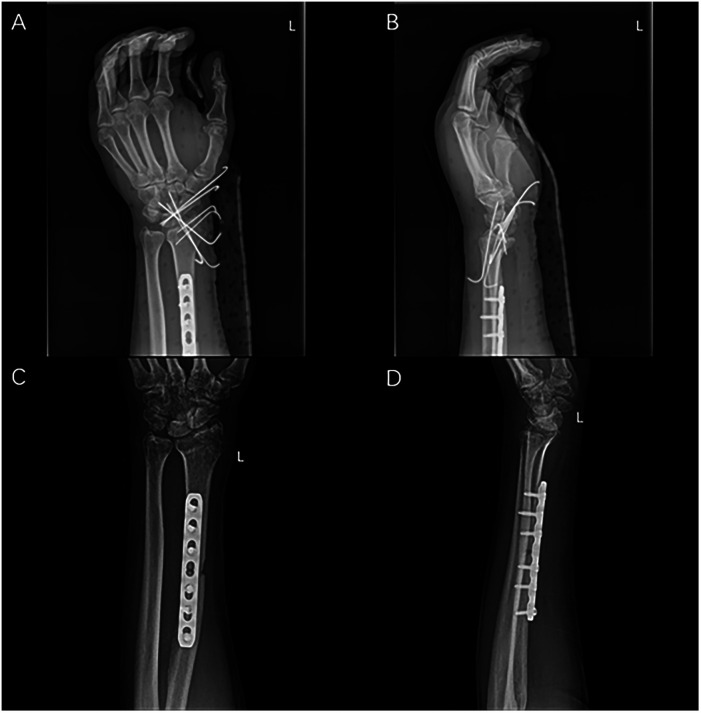
Post-operative radiographs. Anteroposterior radiographs at 3rd day **(A**,**B)** and at 6th week **(C**,**D)**.

Radiographs on 3rd day after surgery manifested the favorable status of reduction and internal fixation. The x-rays at 4th week also showed that the reduction and internal fixation were great, and K-wires were removed at 6th week. The Gilula's carpal arcs were distinct, and the scapholunate interval was normal. (Figure 4) We formulated a personalized rehabilitation plan for this patient to promote the recovery of hand and wrist function, following which the patient attempted less-stress activities at one-month follow-up, though he still complained of mild distending pain.

## 3 Discussion

This article describes a rare case of trans-styloid, palmar-divergent dislocation of the lunate and scaphoid, which was treated with ORIF. In contrast to other 11 cases of palmar-divergent dislocation of the lunate and scaphoid have been reported previously (Table 1), this was the most severe case that it was the first report of trans-styloid palmar-divergent dislocation of the lunate and scaphoid, and nearly all scaphoid- and lunate-related ligaments were disrupted with only residual partial lateral and dorsal ligaments of the scaphoid.

**Table 1:** Review of previous reports with palmar-divergent dislocation of the scaphoid and lunate.

Author	Year	Surgical procedure	Duration of immobilization	Outcomes	Complications
Schroven (3)	2023	Open reduction K-wire pinning	No	No	No
Kao (4)	2020	Closed reduction K-wire pinning	5 weeks	Full motion range	DISI
Phan (19)	2016	Open reduction K-wire pinning	2 months	E: 60° F: 30°	Posttraumatic arthritis
Komura (1)	2011	Closed reduction Anterior capsulodesis Interosseous ligament repair K-wire pinning	7 weeks	E: 50° F: 40°	No
Idrissi (20)	2011	Closed reduction K-wire pinning	6 weeks	E: 40° F: 60°	DISI
Domeshek (21)	2010	Proximal row carpectomy	1 month	No	No
Kang (22)	2003	Open reduction K-wire pinning	6 weeks	Almost full	Intermittent pain
Baulot (23)	1997	Open reduction Anterior capsulodesis	6 weeks	Almost full	DISI
Küpfer (24)	1986	Open reduction K-wire pinning	4 months	E: 25° F: 0°	DISI Avascular necrosis
Cleak (25)	1982	Open reduction Capsulodesis	No	Wrist stiffness	No
Gordon (26)	1972	Open reduction	4 weeks	E: 15° F: 25°	DISI Mild pain

E, extension degree; F, flexion degree; DISI, dorsal intercalated segment instability.

The injury mechanism in this case differs from the classical mechanism due to the specific mode of injury which was applied to the wrist. In Mayfield's classical mechanism, the wrist, in pronated and hyperextended position, suffers high-energy trauma on the palm (6). In contrast, in this case, the patient said that the dorsal carpal was struck by an uncontrollable nozzle of a high-pressure water gun. We have reason to believe that the sudden impact force disrupted the integrity of the proximal row of carpal bones. Meanwhile, the radial styloid and diaphysis fracture also changed the physiological structure of the articular surface on distal radius, which cannot maintain the original position of the lunate and scaphoid.

David W. Meister et al. believed that isolated injury of the SL ligament may not cause carpal instability unless the volar extrinsic ligaments are also disrupted (7). In this case, most ligaments around the proximal row of carpal bones suffered disruption, fortunately, without any carpal bone fractures. During the operation, the palmar radiocarpal ligaments, including the radioscaphoid (RS) ligament, the radioscaph-capitate (RSC) ligament, the long and short radiolunate (RL) ligament, were detected with different degree of injury. In addition, the ulnolunate (UL) ligament was also avulsed. Among the intercarpal internal ligaments, the LT and SL ligaments were identified as completely avulsed. Based on Mayfield's mechanism (6), lunate, without the support of the correlating ligaments, was more easily pushed forward, which was an important cause of its dislocation. Additionally, according to Lauren M Shapiro's research, distal radial fractures or osteotomy would result in increased carpal subluxation (8). Navaratnam et al. also reviewed that lunate dislocation is associated with fractures of the scaphoid and radial styloid (5). Identifying the influence of different status of the radial fracture on the displacement of the scaphoid and lunate is necessary (9–11). Besides, it is essential for exploring the stability of the distal radioulnar joint in this case through finite elements method and cadaveric study for further injury (12). We speculated that increased radial inclination due to diaphysis fracture may contribute to providing larger path for lunate dislocation through the opened space of Poirier. The scaphoid, not completely displaced, benefited from residual dorsal and lateral ligamentous support such as the scaphotrapeziotrapezoid (STT) ligament and had a relatively lower risk of avascular necrosis.

Injury or dissociation of the scapholunate joint is the most common reason of carpal instability. According to current concepts (13), ligament reconstruction is determined by the union status of the lunate and scaphoid to avoid dorsal intercalated segment instability (DISI). In lunate and scaphoid dislocation, three major ligaments containing LT, SL and RSC ligaments may need to be repaired (1, 4). To prevent carpal instability in palmar-divergent dislocation, as the perspectives of Shih-Wen Kao, Shingo Komura and Adnan Kara et al. (1, 4, 14), internal fixation of the SL, LT and RSC joints should be considered to stabilize the entire carpus by ligament repair. Because of that the palmar-divergent dislocation of lunate and scaphoid is extremely unstable, determining how to maximize anatomical carpus reduction and retain the range of motion (ROM) of the wrist is difficult for orthopedic surgeons. In this case, we selected repair of the palmar LT and SL ligaments, anatomic reduction of the SL, RSC and radius-lunate-hamate (RLH) joints with K-wires internal fixation. Different internal fixation methods contribute to different alignment of the carpal bone (4, 15). The avulsed joint capsule and other ligaments would heal gradually during immobilization. Fibrous union without ligament repair cannot maintain carpal stability (16). Although the ligament-reconstruction technique could prevent carpal instability, it also leads to stiffness of the wrist joint (4). Therefore, only interosseous ligaments without extrinsic ligaments were repaired to avoid probable stiffness of the wrist. By this internal fixation, we aimed to strengthen arrangement between the proximal and distal rows to prevent rotation and flexion deformities. Removing K-wires early at the 6th week and rehabilitation is necessary to prevent joint stiffness.

Generally, dorsal palmar approach and combined dorsopalmar approach are recommended for most lunate dislocation. In recent years, closed reduction or arthroscopy has begun to replace some open reductions. Mini incision and arthroscopy appear to result in fewer postoperative complications (17, 18). However, the rigorous indications and technical feasibility limit the application of the minimally invasive surgery, and consensus on this surgery has still not been established.

## 4 Conclusion

In summary, palmar-divergent dislocation of the lunate and scaphoid has a more complicated injury mechanism due to various disruptions around the carpal bones. Standardized management of this injury is immediate reduction and internal fixation, ligament reconstruction, and immobilization with a cast, with the purpose of anatomical reduction and early rehabilitation. The choice of surgical method still depends on the characteristics of the injury and surgeon's preference. The underlying mechanisms necessitate a comprehensive exploration to establish a consensus on how to optimize therapy concepts and minimize postoperative complications.

## Data Availability

The original contributions presented in the study are included in the article/Supplementary Material, further inquiries can be directed to the corresponding author.
